# Acute Dystonia Following Ifosfamide Infusion in a Young Woman with Breast Angiosarcoma: A Case Report

**DOI:** 10.5811/cpcem.62174

**Published:** 2026-09-20

**Authors:** William S. Miller, Alyssa B. Niblett, Charles A. Khoury

**Affiliations:** University of Alabama at Birmingham Heersink School of Medicine, Department of Emergency Medicine, Birmingham, Alabama

**Keywords:** ifosfamide, neurotoxicity, acute dystonia, chemotherapy, emergency medicine

## Abstract

**Introduction:**

Ifosfamide is an alkylating chemotherapeutic agent commonly used in the treatment of sarcomas and other malignancies. Neurotoxicity is a recognized adverse effect, most often presenting as encephalopathy. Acute movement disorders such as dystonia are rare and may be underrecognized in the emergency department.

**Case Report:**

We report a 22-year-old woman receiving combination chemotherapy with doxorubicin, ifosfamide, and mesna for breast angiosarcoma who developed acute right-sided neck spasms and involuntary movements within five minutes of initiating ifosfamide infusion. Vital signs were as follows: temperature, 98.0 degrees Fahrenheit; heart rate, 97 beats per minute; respiratory rate, 18 breaths per minute; blood pressure, 130/88 millimeters of mercury; and oxygen saturation, 99% on room air. Examination revealed sustained contraction of the right sternocleidomastoid and trapezius muscles with involuntary head deviation. She had no exposure to medications commonly associated with dystonia. She was treated with intravenous thiamine, benzodiazepines, intravenous fluids, and baclofen, with subsequent improvement.

**Conclusion:**

Acute dystonia represents a rare manifestation of ifosfamide-induced neurotoxicity. Emergency clinicians should consider chemotherapy-related neurotoxicity in patients presenting with acute movement disorders, as early recognition and supportive management may prevent progression.

## INTRODUCTION

Ifosfamide is an alkylating agent used in chemotherapy for a variety of malignancies, including sarcomas. It is metabolized by hepatic cytochrome P450 enzymes into active metabolites that exert cytotoxic effects through DNA alkylation. Neurotoxicity is a recognized adverse effect, reported in approximately 5%–30% of patients, and most commonly presents as encephalopathy with confusion, hallucinations, or altered mental status.^1^ Movement disorders, including dystonia and myoclonus, are rare manifestations of ifosfamide-induced neurotoxicity and may be unfamiliar to emergency clinicians. With ifosfamide continuing to be used in complex oncologic regimens, emergency physicians may encounter atypical neurotoxic presentations. We describe a case of acute dystonia following ifosfamide infusion to highlight this uncommon but clinically significant adverse effect.

## CASE REPORTS

A 22-year-old woman with right breast angiosarcoma was receiving her first cycle of chemotherapy in our institution’s cancer treatment infusion center. Approximately five minutes after initiation of the ifosfamide infusion, she developed acute headache and involuntary right-sided neck movements and was referred directly to the emergency department for further evaluation. In the emergency department, she reported associated neck pain and limited range of motion. She denied similar prior symptoms, recent illness, trauma, or new medications. This represented the patient’s first cycle of ifosfamide-containing chemotherapy, and she had no prior exposure to ifosfamide.

Vital signs were as follows: temperature, 98.0 °F; heart rate, 97 beats per minute; respiratory rate, 18 breaths per minute; blood pressure, 130/88 mm Hg; and oxygen saturation, 99% on room air. No premedications were administered prior to chemotherapy infusion. The patient had not received dopamine antagonists or other medications commonly associated with acute dystonic reactions, including metoclopramide, prochlorperazine, promethazine, haloperidol, or ondansetron. Examination revealed sustained contraction of the right sternocleidomastoid and trapezius muscles with involuntary head deviation to the right (Image). No focal neurologic deficits were present.

The differential diagnosis included acute drug-induced dystonia, seizure, cervical muscle spasm, and acute cerebrovascular event. The close temporal relationship to ifosfamide infusion and isolated focal dystonia supported a medication-induced etiology. The patient was treated with intravenous thiamine and benzodiazepines, with moderate improvement. She was admitted for further evaluation, including electroencephalography and brain magnetic resonance imaging. Her presentation was attributed to ifosfamide-induced acute dystonia.

## DISCUSSION

Ifosfamide-induced neurotoxicity includes a spectrum of neurologic complications ranging from mild confusion to severe encephalopathy, hallucinations, seizures, and movement disorders. Encephalopathy is the most frequently reported manifestation; movement abnormalities such as dystonia are rare. Toxic metabolites, including chloroacetaldehyde, are thought to contribute through mitochondrial dysfunction and glutathione depletion.^2^–^4^

A formal adverse drug reaction assessment using the Naranjo Adverse Drug Reaction Probability Scale yielded a score of 6, consistent with a probable adverse drug reaction. The rapid onset of symptoms following initiation of ifosfamide infusion, absence of alternative etiologies, objective examination findings, and subsequent clinical improvement support a causal relationship. For emergency clinicians, recognition of this entity is important to avoid unnecessary diagnostic testing and to facilitate early targeted management.

*CPC-EM Capsule*

What do we already know about this clinical entity?

*Ifosfamide is associated with neurotoxicity, most commonly presenting as encephalopathy.*

What makes this presentation of disease reportable?

*Acute dystonia is a rare and underrecognized manifestation of ifosfamide-induced neurotoxicity.*

What is the major learning point?

*Acute dystonia can represent ifosfamide-induced neurotoxicity and may occur shortly after infusion.*

How might this improve emergency medicine practice?

*Early recognition and supportive treatment, including consideration of thiamine, may prevent progression of neurotoxicity.*

Thiamine has been reported as a therapeutic adjunct in ifosfamide-induced neurotoxicity and may improve neurologic symptoms.^2^,^5^ Although evidence is limited, thiamine is low risk and may be considered in suspected cases. Management centers on discontinuation or dose adjustment of ifosfamide, supportive care, and symptomatic treatment with benzodiazepines or antispasmodics. Early recognition may prevent progression to more severe neurologic complications.

## CONCLUSION

Acute dystonia is a rare manifestation of ifosfamide-induced neurotoxicity. Emergency clinicians should maintain a high index of suspicion for chemotherapy-related neurotoxicity in patients presenting with acute movement disorders, as early recognition and supportive management may improve outcomes.

## Figures and Tables

**Image:**
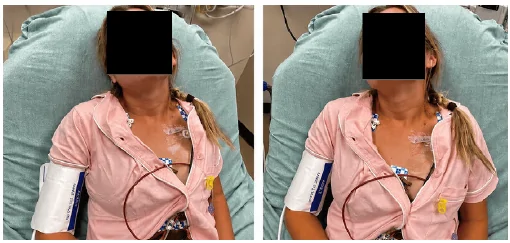
Acute cervical dystonia demonstrating sustained involuntary contraction of the right sternocleidomastoid and trapezius muscles with resultant rightward head deviation following ifosfamide infusion.
